# Laboratory colonization and maintenance of *Anopheles atroparvus* from the Ebro Delta, Spain

**DOI:** 10.1186/s13071-020-04268-y

**Published:** 2020-08-03

**Authors:** Lotty Birnberg, Carles Aranda, Sandra Talavera, Ana I. Núñez, Raúl Escosa, Núria Busquets

**Affiliations:** 1 Centre de Recerca en Sanitat Animal (CReSA), Institut de recerca en Tecnologies Agroalimentaries (IRTA), Barcelona, Spain; 2 Servei de Control de Mosquits del Consell Comarcal del Baix Llobregat, Barcelona, Spain; 3 Consorci de Polítiques Ambientals de les Terres de lʼEbre (COPATE), Tarragona, Spain

**Keywords:** *Anopheles atroparvus*, Colonization, Malaria, Europe

## Abstract

**Background:**

Historically, *Anopheles atroparvus* has been considered one of the most important malaria vectors in Europe. Since malaria was eradicated from the European continent, the interest in studying its vectors reduced significantly. Currently, to better assess the potential risk of malaria resurgence on the continent, there is a growing need to update the data on susceptibility of indigenous *Anopheles* populations to imported *Plasmodium* species. In order to do this, as a first step, an adequate laboratory colony of *An. atroparvus* is needed.

**Methods:**

*Anopheles atroparvus* mosquitoes were captured in rice fields from the Ebro Delta (Spain). Field-caught specimens were maintained in the laboratory under simulated field-summer conditions. Adult females were artificially blood-fed on fresh whole rabbit blood for oviposition. First- to fourth-instar larvae were fed on pulverized fish and turtle food. Adults were maintained with a 10% sucrose solution *ad libitum*.

**Results:**

An *An. atroparvus* population from the Ebro Delta was successfully established in the laboratory. During the colonization process, feeding and hatching rates increased, while a reduction in larval mortality rate was observed.

**Conclusions:**

The present study provides a detailed rearing and maintenance protocol for *An. atroparvus* and a publicly available reference mosquito strain within the INFRAVEC2 project for further research studies involving vector-parasite interactions. 
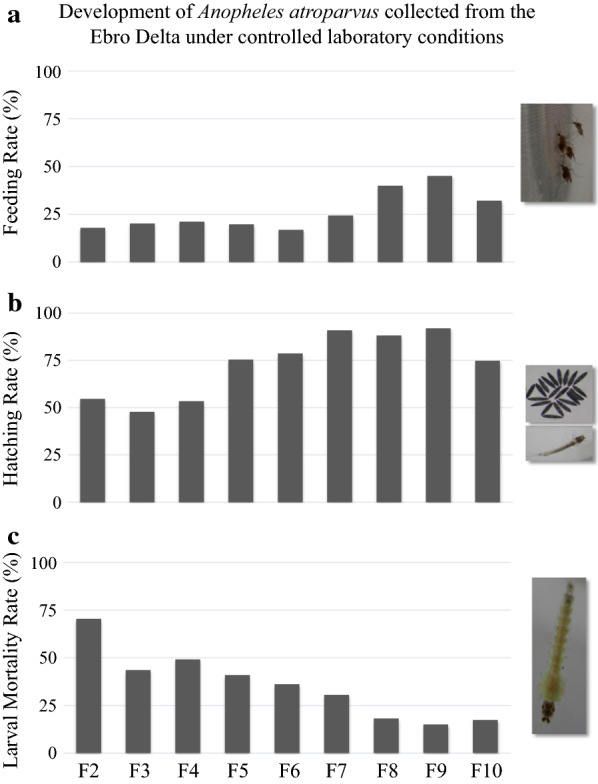

## Background

In Europe and the Middle East, dominant *Anopheles* vector species primarily belong to the *Anopheles maculipennis* subgroup [1]. Among its 11 Palaearctic sibling species [2, 3], *An. atroparvus* (van Thiel, 1927), is the most abundant and widely distributed [4]. This species inhabits coastal and inland areas throughout eastern and central Europe, the Iberian Peninsula and the UK [1, 5]. However, its absence has been suggested in Greece, Turkey [6] and partially in southern Italy where it is replaced in coastal areas by *An. lanbranchiae* [7]. Immature stages of *An. atroparvus* mostly inhabit a variety of permanent or semi-permanent water bodies characterized by clear standing, or slow flowing, brackish and/or fresh water. They are commonly collected along river and lake margins, marshes, irrigation canals and especially in rice fields (primary larval habitat), where aquatic vegetation provides protection from predators and a cooler environment [8, 9]. *Anopheles atroparvus* has been described as an endophilic, most commonly endophagic, and zoophilic species with a marked preference for domestic farm animals [10–14]. Due to its association to human settlements, *An. atroparvus* also demonstrates anthropophilic behavior [1].

Historically, *An. atroparvus* was implicated in the transmission of local strains of both *Plasmodium vivax* [9] and *P. falciparum* [8]. A recent study in which DNA was recovered from historic blood slides of patients infected during the 40’s showed that both *P. vivax* and *P. falciparum* were circulating at Ebro Delta (Spain) [15], an area where *An. atroparvus* is the only anopheline species recorded [15, 16]. Moreover, susceptibility tests demonstrated that different European populations were capable of transmitting imported *P. vivax* [17] and *P. ovale* strains [18], but were, to some degree, refractory to tropical *P. falciparum* strains [17, 19, 20].

Currently, despite the situation that most of the European continent demonstrates “anophelism without malaria” [8], significant increases in the number of imported cases [5], sporadic episodes of local transmission in some countries [21–27], and predictions that climatic change could increase the risk of malaria transmission [4, 9, 28, 29] have raised new concerns for the re-introduction of malaria.

To better assess the potential risk of malaria resurgence in Europe, it is necessary to conduct vector competence studies to establish the vector-parasite relationships between local populations of *Anopheles* mosquitoes with the most commonly imported *Plasmodium* species. Consequently, as a first step, the aim of the present study was to establish a laboratory colony of *An. atroparvus* from the Ebro Delta, a former malaria endemic area of Spain, and provide a detailed rearing protocol for further malaria research.

## Methods

### Study area

The Ebro Delta is one of the most relevant ecosystems in the Western Mediterranean. It is located in Tarragona Province (Catalonia-Spain) and covers 320 square kilometers. The Ebro River divides the delta plain into two regions, the Baix Ebre from the north, with its capital Tortosa; and the Montsià from the south, with its capital Amposta. The delta is characterized by highly diverse aquatic habitats, e.g. marshes, wetlands, ponds and lakes that co-occur with densely populated areas and croplands, mostly intended for rice cultivation. The dominance of water systems in the Ebro Delta have favored the proliferation of vector mosquito species, e.g. *An. atroparvus* which was previously incriminated as a primary malaria vector [28].

### Field mosquito collections

To start the laboratory colony, adult anopheline mosquitoes were collected weekly between August and September 2017. In rice growing areas from the municipality of Amposta (40°42′32.5686″N, 0°35′12.2814″E), resting male and female mosquitoes were collected in an unused shed using mouth aspirators (John W. Hock Company, Gainesville, FL, USA), placed in 30 × 30 × 30 cm BugDorm (Bioquip, Rancho Dominguez, CA, USA) insect rearing cages and transported live to the laboratory.

### Laboratory mosquito rearing protocol

At the Institut de Recerca i Tecnologies Agroalimentaries - Centre de Recerca en Sanitat Animal (IRTA-CReSA) biosafety level 2 facilities (BSL2), a sterile 10% sucrose solution was provided to wild-caught adults by placing a 50 ml glass bottle of the solution containing a filter paper fan for mosquitoes to feed *ad libitum*. Ten percent (10/100) of the captured females were dissected to determine gravid rates. Since all the dissected females were gravid, a Petri dish filled with dechlorinated tap water was placed inside the cages for oviposition. Since no eggs were laid during the first week, several artificial blood meals were offered. Field-collected females were provided blood meals on fresh whole rabbit blood (supplied by a local slaughterhouse) for 3 h at dusk using the Hemotek feeding system (Discovery Workshop, Accrington, UK) set at 37.5 ± 0.5 °C and Parafilm as a feeding membrane. On day 1 post-feeding, a Petri dish containing dechlorinated tap water for oviposition was placed inside the cages and kept until eggs were laid. Egg batches were transferred to sterile plastic trays (22 × 15 × 6 cm) containing 500 ml of dechlorinated and oxygenated tap water. One-fourth Gayelord Hauser Superlevure brewer’s yeast tablet was added to stimulate hatching. To confirm the identity of this mosquito population, 25 wild-caught females (that fed and oviposited) were molecularly analyzed by polymerase chain reaction (PCR) [30].

Upon hatching, up to 100 first-instar larvae (L1) were transferred to sterile plastic trays (22 × 15 × 6 cm) containing 500 ml of dechlorinated and oxygenated tap water. Larvae (L1 to L4), were fed 0.1 g minced Tetra Goldfish Flakes and Tetra ReptoMin Sticks (1:1) mixture. Water from rearing trays and food supply were replaced daily.

Pupae were collected daily using a 3 ml plastic pipette and deposited in sterile plastic cups (9 cm in diameter per 7 cm height) containing dechlorinated and oxygenated tap water. Cups containing F1 pupae, were placed inside 30 × 30 × 30 cm BugDorm (Bioquip) insect cages with a density of 500 specimens per cage. Adults were provided a 10% sucrose solution *ad libitum* as previously described.

Rearing procedures were followed for subsequent generations with slight modifications: (i) ten day-old (or older) females were deprived sucrose for 48 h and provided blood meals as described above, blood-fed females were placed in a separate cage after feeding; (ii) the oviposition Petri dish with dechlorinated tap water was placed in the cage containing blood-fed mosquitoes at day 5 post-feeding; and (iii) water from larval trays was replaced every 2 days during development. The day the water was not changed, 100 ml of oxygenated and dechlorinated tap water was added to oxygenate and maintain water level. Larval food was added daily.

The life-cycle of *An. atroparvus* mosquitoes was monitored under controlled laboratory conditions simulating field summer conditions of their original habitat (temperature: 25–20 °C for day and night respectively, relative humidity: 80%, and a photoperiod: 12 h light: 11 h dark with two 30 min crepuscular periods).

### Colony assessment

Hatching, larval mortality and feeding rates were calculated and, larval and pupal development times were determined to evaluate laboratory adaptation of the colony. The hatching rate (HR) was calculated as the proportion of L1 larvae/number of eggs. Larval mortality rate (LMR) was calculated as the total number of pupae/L1 larvae. Feeding rate (FR) was calculated as the number of engorged females/the total number of females at the time of blood-feeding. Larval and pupal development times were calculated, respectively, as the number of days between L1 to pupae, and from pupae to adult emergence. Since most comprehensive data were obtained from the second generation (F2), hatching, feeding and mortality rates were calculated from this time point onwards. For larval and pupal development times, data from the fourth generation (F4) onwards were used. The purity of the colony was molecularly verified by PCR [30] analyzing 10 females from both, F6 and F10.

## Results and discussion

An indigenous *An. atroparvus* population from Amposta (the Ebro Delta) was successfully colonized in our laboratory and its rearing protocol standardized. The colony constitutes one of the reference mosquito strains available within the INFRAVEC2 project for vector research.

**Fig. 1:**
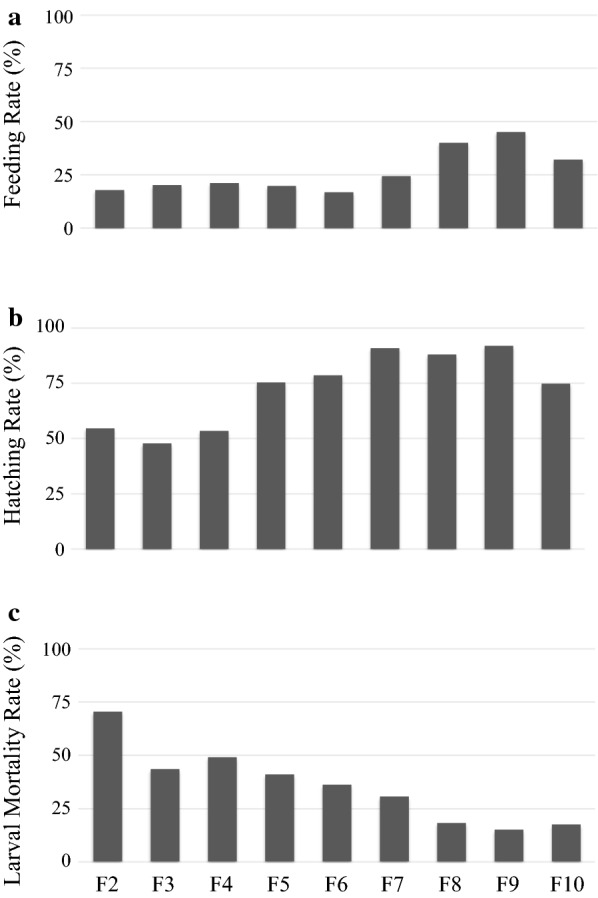
Development of *Anopheles atroparvus* collected from the Ebro Delta under controlled laboratory conditions. **a** Feeding rate (FR), engorged females/total number of females at the time of feeding. **b** Hatching rate (HR), total number of L1/total number of eggs. **c** Larval mortality rate (LMR), total number of pupae/total number of L1

Approximately 20% of 10-day-old females from generations F2-F6 fed on rabbit blood provided by an artificial (Parafilm) membrane. However, the feeding rates increased up to 45% in later generations (F9) (Fig. 1a). Eggs were oviposited on day 5 post-blood feeding and eggs hatched after 1–2 days. In early generations (F2-F4), between 48–55% of the eggs hatched, while in later generations, hatching rates increased to 75–92% (Fig. 1b). The increase in hatching rates reflects the successful adaptation of male-mating activity as reported for other free-mating culicids [31, 32].

Throughout laboratory colonization, a clear reduction in larval mortality was observed, from 70% in F2, to less than 20% in the latest generations (Fig. 1c), with more than 80% of the first instars reared to adults. The mortality of pupae was almost null in all generations. Both, larval and pupal development times were variable. On average, 13–16 days were required from L1 to pupae (larval development time), and between 1–3 days from pupae to adult emergence (pupal development time) (Table 1). A 1:1.14 female:male ratio was observed. Adult lifespan in our laboratory under field-simulated conditions surpassed nine weeks, enough time to conduct vector competence and susceptibility assays. The stenogamic behavior described for Spanish populations [13] was confirmed in the *An. atroparvus* colony and under laboratory conditions males successfully mated with females in small cages. Swarming and mating events were observed during blood feeding, contradicting previous behavioral descriptions [8]. Egg development and development times of immature stages observed under the present laboratory conditions were in agreement with previous studies that used similar temperatures [8], showing the relevance of this variable during colonization attempts of vector mosquito species.

**Table 1:** Development times in immature stages of *Anopheles atroparvus* during laboratory colonization

Generation	Development time (days)	
	Larva to pupa	Pupa to adult
F2	9–25	na
F3	9–25	na
F4	8–22	1–3
F5	12–25	1–4
F6	11–24	1–4
F7	10–26	2–4
F8	8–22	1–3
F9	10–21	1–3
F10	10–18	1–3

*Abbreviation*: na, data not available

Finally, that the diagnostic PCR methods described by Proft et al. [30] for the identification of six sibling species of the Maculipennis subgroup resulted in the amplification of three fragments per individual, which corresponded in size to *An. atroparvus* (117 bp), *An. melanoon* (224 bp) and *An. labranchiae* (374 bp). However, after sequencing, all three PCR products corresponded to gene sequences of *An. atroparvus*. Based on our experience [16], *An. atroparvus* is the only anopheline species distributed in this area and these findings suggest that the single 3’-end nucleotide substitution in the primer annealing sites, in the case of *An. melanoon* and *An. labranchiae*, does not provide a unique diagnostic gene fragment for the *An. atroparvus* population studied.

## Conclusions

The present study provides a detailed protocol used to successfully establish and maintain a laboratory colony of a European strain of *An. atroparvus*. Field-caught specimens were only fed *via* artificial membrane feedings, facilitating the logistics during colony maintenance and during vector competence studies. The potential to evaluate pathogen susceptibility using artificial blood-feeding techniques of earlier laboratory generations would provide a more accurate assessment of vector competence of wild populations.

## Data Availability

All data generated or analyzed during this study are included in the article.

## Abbreviations

HR: hatching rate
LMR: larval mortality rate
FR: feeding rate
L1: first-instar larva
L2: second-instar larva
L4: fourth-instar larva
